# Beyond Infection: Mitochondrial Reprogramming and Immunometabolic Adaptation in *Helicobacter pylori*-Associated Gastric MALT Lymphoma

**DOI:** 10.3390/diseases14080280

**Published:** 2026-08-05

**Authors:** Ciro Gargiulo Isacco, Van Hung Pham, Huong Thien Pham, Kieu Cao Diem Nguyen, Toai Cong Tran, Thach Huy Le, Felicita Jirillo, Emilio Jirillo, Luigi Santacroce

**Affiliations:** 1 Department of Interdisciplinary Medicine, Section of Microbiology and Virology, School of Medicine, University of Bari “Aldo Moro”, 70124 Bari, Italy; drkieukaren@humanstemcell.net (K.C.D.N.); emilio.jirillo@uniba.it (E.J.); luigi.santacroce@uniba.it (L.S.); 2 Viet Nam Research and Development Institute of Clinical Microbiology, Ho Chi Minh City 756000, Vietnam; phhvan.nkbiotek@gmail.com (V.H.P.); phamthienhuong@gmail.com (H.T.P.); 3 Basic Medical Sciences Faculty, Pham Ngoc Thach University of Medicine, Ho Chi Minh City 740500, Vietnam; trancongtoai@pnt.edu.vn; 4 Ninh Thuan General Hospital, Khanh Hoa City 59123, Vietnam; lh.thach67@gmail.com; 5 Independent Researcher, R&D, 70124 Bari, Italy; felicitajirillo@hotmail.com

**Keywords:** *Helicobacter pylori*, antibiotic therapy, B cells, mitochondria, gastric mucosa-associated lymphoid tissue lymphoma (MALT)-(GML/MALToma), gastric B-cell large lymphoma (BCLL), cytotoxin-associated gene A (CagA), vacuolar cytotoxin A (VacA)

## Abstract

Gastric mucosa-associated lymphoid tissue (MALT) lymphoma, also known clinically as gastric MALT lymphoma (GML) or MALToma, is an indolent B-cell neoplasm strongly associated with chronic Helicobacter pylori (*H. pylori*) infection. While early-stage disease is based on persistent antigenic stimulation and chronic inflammation, the metabolic and molecular transitions that drive monoclonal B-cell autonomy remain poorly understood. Importantly, *H. pylori* maintain this long-term colonization by defusing the host’s innate immunity; specifically, its lipid A portion features unique elongated acyl chains, composed of 16–18 carbon atoms, that fail to bind to and activate host TLR4/MD2 receptors, resulting in exceptionally weak endotoxic potency. Persistent colonization relies on key oncoproteins, particularly cytotoxin-associated gene A (CagA) and vacuolar cytotoxin A (VacA), which orchestrate early inflammatory infiltration (neutrophils, Th1, Th2 and Th17 cells) before shifting the microenvironment toward a suppressive regulatory T cell (Treg) phenotype. In this study, we propose a new critical step in the oncogenesis of gastric metastasis: chronic mitochondrial and immunometabolic adaptation within the gastric microenvironment. We claim that *H. pylori* act not only as a trigger for infection but also as a chronic driver of mitochondrial adaptation to oxidative stress and hypoxia, which subsequently results in defective mitophagy. CagA- and VacA-mediated mitochondrial damage induces reactive oxygen species (ROS) and functional hypoxia, stabilizing HIF-1α to force a glycolytic metabolic shift, while incomplete mitophagy rescues metabolically altered, apoptosis-resistant clones to drive monoclonal B-cell expansion. Within this ecological-microenvironmental framework, the predominantly cytoplasmic sequestration of BCL10 and the NF-κB subunit p65 observed in GML is reinterpreted not as evidence of signaling inactivity, but as a dynamically regulated adaptive state. This configuration is orchestrated by mitochondrial stress responses that enable adaptation to the chronic microenvironmental pressures imposed by *H. pylori*, acting in concert with the metabolic programs governed by MYC, NRF2, and BCL2. Overall, this review outlines the multi-step pathogenesis of *H. pylori*-mediated GML, highlighting how mitochondrial dysfunction and metabolic remodeling drive the transition from chronic infection to malignant transformation.

## 1. Introduction

*H. pylori* have been classified as a class I carcinogen worldwide in view of its primary involvement in the development of Gastric mucosa-associated lymphoid tissue (MALT) lymphoma (GML/MALToma), an indolent, non-Hodgkin lymphoma, originates from memory B cells located in the marginal zone of secondary lymphoid tissues [1]. In general, MALT lymphomas have different localizations, such as the stomach (34%), ocular adnexa (13%), lungs (8.8%), salivary glands (8.3%), the colon rectum (5.2%), and the small intestine (3.4%) [2]. GML occurs in patients with a mean age of 60 years at the time of diagnosis and is more prevalent in men than in women [3]. From a genetic point of view, in the development of GML, the most common translocation is represented by t(11;18) (q21; q21) with a fusion between the N-terminus of the API-r gene and the C-terminus of the MALT-I gene [3]. Such an event leads to the activation of NF-kB, which regulates genes involved in lymphocyte proliferation and survival [3]. Moreover, the above mutation decreases the antibiotic response against *H. pylori*, but with a limited risk of transformation into a more aggressive gastric B-cell large lymphoma (BCLL) [3,4]. Histologically, GML is composed of heterogeneous small B cells, including centrocyte-like cells, monocytic cells, small lymphocytes, immunoblastic cells, and centroblast-like cells, with a proportion of cases characterized by plasma cell differentiation [4]. GML is linked with B Cell markers (CD19+, CD20+ and CD79a+), LN-1+, CD5-, CD10-, CD23-, IgD-, IgM (>IgA>IgG), Bcl6-, and cyclin D1 [5].

From a pathogenetic point of view, MALT lymphomas are associated with persistent chronic stimulation by bacterial or viral antigens, autoimmunity (Sjogren syndrome or Hashimoto’s thyroiditis), or IgG4-related disease [6]. In this respect, the bacterial etiopathogenesis of GML is supported by its association with *H. pylori* infection [7]. However, other bacteria have been implicated in the pathogenesis of MALT lymphomas, such as *Borrelia burgdorferi* (in the skin), *Chlamydophila psittaci* (in the ocular adnexa), *Achromobacter xylosoxidans* (in the lung), and *Campylobacter jejuni* (in the small intestine) [8]. Of note, there is also evidence of *H. pylori*-negative GML, and ongoing research is aimed at uncovering the potential factors contributing to its development, as discussed in the next sections of this review [9]. Nevertheless, among the microbial factors associated with GML, *H. pylori* remain the predominant etiological agent, accounting for approximately 90% of cases, and persistent colonization creates a chronic antigen-driven inflammatory environment in which continuous exposure to bacterial antigens and autoantigens promotes sustained B-cell activation and proliferation [9,10]. This prolonged immune stimulation supports the formation of organized lymphoid tissue within the gastric mucosa and creates conditions conducive to uncontrolled clonal B-cell expansion that over time may drive lymphoid transformation, ultimately leading to the development of gastric MALT lymphoma [10].

*H. pylori* is a Gram-negative bacterium that colonizes the gastric mucosa in approximately 50% of the world’s population [11]. Its presence is associated with gastritis, duodenal ulcer disease, and GML, while chronic infection can cause persistent and progressive damage to the gastric mucosa [12]. Despite repeated eradication treatments, *H. pylori* may persist or recur, reflecting its remarkable ability to adapt to the hostile gastric environment and evade host immunosurveillance [13]. A key determinant of its persistence is urease production, which hydrolyzes urea and generates ammonia, buffering gastric acidity and enabling bacterial survival within the low-pH gastric environment [14].

*H. pylori* use its flagella to penetrate the gastric mucosa layer and reach the epithelial surface, where specific adhesins mediate attachment to host–cell receptors and facilitate stable colonization [15]. These adhesion factors include blood group antigen-binding adhesin (BabA), sialic acid-binding adhesin (SabA), and outer inflammatory protein A (OipA) [15,16]. *H. pylori* also produce two major virulence factors, cytotoxin-associated gene A (CagA) and vacuolating cytotoxin A (VacA), which exert distinct effects on gastric epithelial and immune cells. CagA, delivered into host cells through the type IV secretion system, disrupts intracellular signaling, cell polarity, and epithelial homeostasis, whereas VacA alters membrane integrity, intracellular trafficking, and immune-cell function. These factors promote persistent mucosal inflammation, impair protective immune responses, and contribute to the cellular and molecular alterations associated with gastric carcinogenesis [15,16,17] (Table 1).

Quite importantly, another factor contributing to *H. pylori*’s immune escape is represented by its lipopolysaccharide (LPS) [18]. Unlike the lipid A of most Gram-negative bacteria, which typically consists of a hexa-acylated structure containing six fatty acyl chains of 12–14 carbon atoms, *H. pylori* synthesize a unique tetra-acylated lipid A composed of only four fatty acyl chains, each unusually long. Together with the removal of the 4′-phosphate group, this distinctive structural remodeling profoundly alters the conformation and electrostatic properties of lipid A [19]. In this respect, Lipid A leads to weak activation of innate receptors, e.g., Toll-like Receptors (TLRs), located on gastric epithelial and immune cells, thus dampening the host protective immune response, as a further escape mechanism [20]. However, there is evidence that *H. pylori*, despite its low endotoxic potency, can elicit a biphasic immune response. At the initial stage of infection, *H. pylori* recruit gastric mucosa’s innate immune cells, i.e., neutrophils, monocytes, dendritic cells (DCs) and macrophages, with the release of pro-inflammatory cytokines and chemokines [21]. Beyond front-line innate evasion, long-term gastric colonization is sustained by a shift in the adaptive immune system toward active tolerance. While localized Th17 or transient Th2 responses may occur, chronic infection is primarily driven by the expansion of regulatory T cells (Tregs), macrophages (from type 1 to type 2) and dendritic cell (DCs) reprogramming [21]. The emergence of M2-polarized macrophages profoundly influences B-cell biology. Together with DCs, Tregs and activated T-helper lymphocytes, these cells establish a cytokine-rich microenvironment that sustains antigen-dependent B-cell proliferation through CD40-CD40L interactions and the expression of the co-stimulatory molecules CD80 and CD86 [22,23]. Simultaneously, activation of the non-canonical NF-κB pathway stimulates the production of B-cell activating factor (BAFF) and a proliferation-inducing ligand (APRIL), two essential mediators of B-cell survival, differentiation, and immunoglobulin production [23]. Continuous exposure to these signals promotes the formation of ectopic lymphoid follicles within the gastric mucosa, creating the anatomical and immunological substrate from which GML ultimately arises in this tolerogenic milieu, reprogrammed DCs downregulate IL-12 production and enhance the release of immunoregulatory cytokines, particularly IL-10 and TGF-β [23]. Through sustained IL-10, M2 and TGF-β-mediated suppression of effector responses, Tregs limit protective antibacterial immunity, allowing *H. pylori* to establish lifetime colonization [24]. Downregulation of the immune response is a crucial part of invading strategy based on anti-tumor cytotoxic T cell function, that promotes oncogenesis. In this review, the mechanisms elicited by *H. pylori* infection in terms of persistent gastric inflammation and the development of GML will be illustrated, with particular emphasis on the role of mitochondrial reprogramming and the emergence of B cells capable of avoiding apoptosis through alterations in selected mitochondria-regulated apoptotic pathways [23]. With special reference to the *H. pylori*-mediated B cell response, the O-polysaccharide chain of *H. pylori* LPS is highly fucosylated and structurally mimics human Lewis antigens, enhancing the production of anti-Lewis antibodies, which may subsequently trigger the expansion of autoreactive B cell clones [24]. The *H. pylori* development of GML will be discussed to elucidate the pathogenesis of this type of lymphoma.

## 2. Gastric MALT Lymphoma: Diagnosis, Treatment, and Molecular Mechanisms of Disease Progression

GML is characterized by non-specific upper gastrointestinal symptoms, with endoscopy, biopsies, and immunochemistry as golden diagnostic standards. Major histological hallmarks are represented by lymphoepithelial lesions with destruction of gastric glands or pits, and evidence of centrocyte-like cells, and reactive germinal centers [25]. Patients with *H. pylori*-positive GML should receive *H. pylori* eradication therapy as the initial therapeutic approach. Bismuth quadruple therapy (BQT) is currently among the preferred first-line regimens, particularly in settings where clarithromycin resistance is prevalent or susceptibility testing is unavailable [26].

This treatment combines a proton pump inhibitor (PPI), bismuth, tetracycline, and metronidazole, hence partially overcoming the growing problem of antimicrobial resistance and achieving high eradication rates. For these reasons, the most recent international guidelines recommend optimized BQT as the empirical treatment of choice in many geographical areas, typically administered for 14 days [26]. Histological regression occurs in the first 2 to 6 months; in some cases, recurrence can appear in *H. pylori* eradicated lymphoma even after remission. In patients who do not respond to eradication therapy, radiotherapy and, in more advanced or refractory cases, systemic chemo-immunotherapy become necessary [27]. With special reference to *H. pylori*-negative GML, its incidence is increasing, reaching about 40% of all GML [28]. According to Gu and colleagues [29], the very large use or abuse of antibiotics may result in a decreased prevalence of *H. pylori* infections in Western countries. On the other hand, other species of *H. pylori*, e.g., *H. heilmannii*, have been detected in patients with GML, and this fact may explain why a small subset of *H. pylori*-negative GML, without background gastritis, responds to antibiotic therapy [30].

However, the National Comprehensive Cancer Network guidelines recommend radiotherapy for patients with *H. pylori*-negative gastric GML, with only a limited role for antibiotic therapy [31]. Progression of *H. pylori*-positive GML to diffuse large B-cell lymphoma (DLBCL) has also been reported. Interestingly, *H. pylori*-positive DLBCL appears to have a more favorable prognosis than its *H. pylori*-negative counterpart [32]. This protective effect has been associated with the inhibition of ZEB1 mediated by increased expression of miRNA-200/210, together with higher levels of BCL-6 expression [33]. Within this molecular network, mitochondria start emerging as central metabolic regulators and stress sensors that influence cell-fate decisions, including survival, epithelial–mesenchymal transition (EMT), differentiation, and neoplastic progression through MYC and BCL protein family [34].

While MYC is a master regulator of cell-cycle progression, biomass accumulation, and rapid cellular proliferation, members of the BCL-2 protein family critically regulate mitochondrial outer membrane permeability and the intrinsic apoptotic pathway [35]. Importantly, within this context, mitochondria are not merely passive targets of oncogenic signaling but active metabolic regulators capable of modulating MYC expression through retrograde signaling, metabolic feedback loops, ROS, and stress-responsive miRNA networks [35,36]. Under metabolic stress, mitochondrial crosstalk sustains MYC activity to fuel cell proliferation and metabolic reprogramming [37]. Concurrently, BCL-2 maintains outer membrane integrity to block pro-apoptotic mediators like cytochrome c, shielding lymphoma cells from cell death [37]. This combination of MYC-driven growth and BCL-2 survival signaling creates an optimal environment for malignant transformation and disease progression [38,39].

As genomic instability accumulates during lymphoma progression, additional molecular determinants become involved. Among these, mitotic arrest deficiency protein 2 (MAD2), a key component of the spindle assembly checkpoint, which plays a critical role in maintaining chromosomal stability during mitosis. Overexpression of MAD2 in GML and DLBCL has been associated with reduced disease-free survival and may reflect increased chromosomal instability, enhanced proliferative capacity, and a greater predisposition for transformation toward more aggressive lymphoma phenotypes [40]. Furthermore, the coexistence of MAD2-driven genomic instability and mitochondria-mediated adaptive responses may ease the selection and survival of genetically altered lymphoma clones. By supporting cellular strength under conditions of replication stress, metabolic imbalance, and DNA damage, these interconnected pathways may contribute to lymphoma progression, therapeutic resistance, and disease persistence [40,41].

## 3. Virulence Factors and Antigenic Determinants of *H. pylori* in Gastric MALT Lymphomagenesis

The initial adhesion of *H. pylori* to the gastric epithelium is subsequently reinforced by biofilm formation within an extracellular matrix, enhancing bacterial persistence and protecting the pathogen from host immune-mediated clearance [11,12,13].

To extend its pathological reach beyond immediate cellular attachment, *H. pylori* release outer membrane vesicles (OMVs) containing key virulence factors, including lipopolysaccharide (LPS), CagA, VacA, adhesins, urease, catalase, and serine proteases [42]. These OMVs facilitate the long-distance delivery of bacterial components across the gastric microenvironment, shielding *H. pylori* from host immune defenses and antimicrobial agents while simultaneously promoting chronic inflammation and local immune suppression [42,43] (Table 1).

The main intent is to establish a “tolerogenic settlement” within the gastric mucosa, *H. pylori* strategically alter its surface structures to sabotage pattern-recognition receptors (PRRs), particularly TLR4 and TLR5. Expressed on epithelial cells, DCs, and macrophages, these transmembrane receptors normally recognize microbial components to initiate NF-κB activation and pro-inflammatory cytokine production [44]. *H. pylori* evade this surveillance by structurally modifying its flagellin to avoid recognition by TLR5 [44]. Similarly, its Lipid A undergoes specific modifications that markedly reduce its endotoxic activity and diminish its capacity to activate the TLR4/MD-2 receptor complex [45,46]. Of course, *H. pylori* LPS is significantly less endotoxic than that of *Salmonella minnesota* or *Escherichia coli* [46]. From an oncogenic standpoint, this modified Lipid A reduces natural killer (NK) cell activation and promotes the expansion of Tregs, thus dampening host antitumor immune responses [47].

In parallel, *H. pylori* employ molecular mimicry through its highly conserved, extensively fucosylated O-polysaccharide antigen. This structure closely mimics human Lewis blood-group antigens (${\mathrm{Le}}^{x}$, ${\mathrm{Le}}^{y}$, ${\mathrm{Le}}^{a}$, ${\mathrm{Le}}^{b}$, ${\mathrm{Le}}^{c}$, and sialyl-Lewis antigens) as well as the H-1 antigen [48]. Recognized in part by the gastric lectin galectin-3, this molecular camouflage facilitates host–pathogen interactions and enables the bacteria to evade immune recognition, fostering long-term colonization [48,49,50]. However, this structural mimicry also triggers the production of autoreactive antibodies and chronic immune stimulation [50].

The pathogenic capacity of *H. pylori* is further driven by specific oncogenic effectors. Approximately 70% of *H. pylori* strains harbor the CagA protein, which is injected directly into host epithelial cells via a type IV secretion system (T4SS) following interaction with $\beta_{1}$ integrins [51,52,53]. Concurrently, *H. pylori* secrete Vac-A responsible for membrane pore formation and a C-terminal p55 domain mediating cell surface receptor binding and endocytosis [54,55,56] (Table 2).

**Figure 1:**
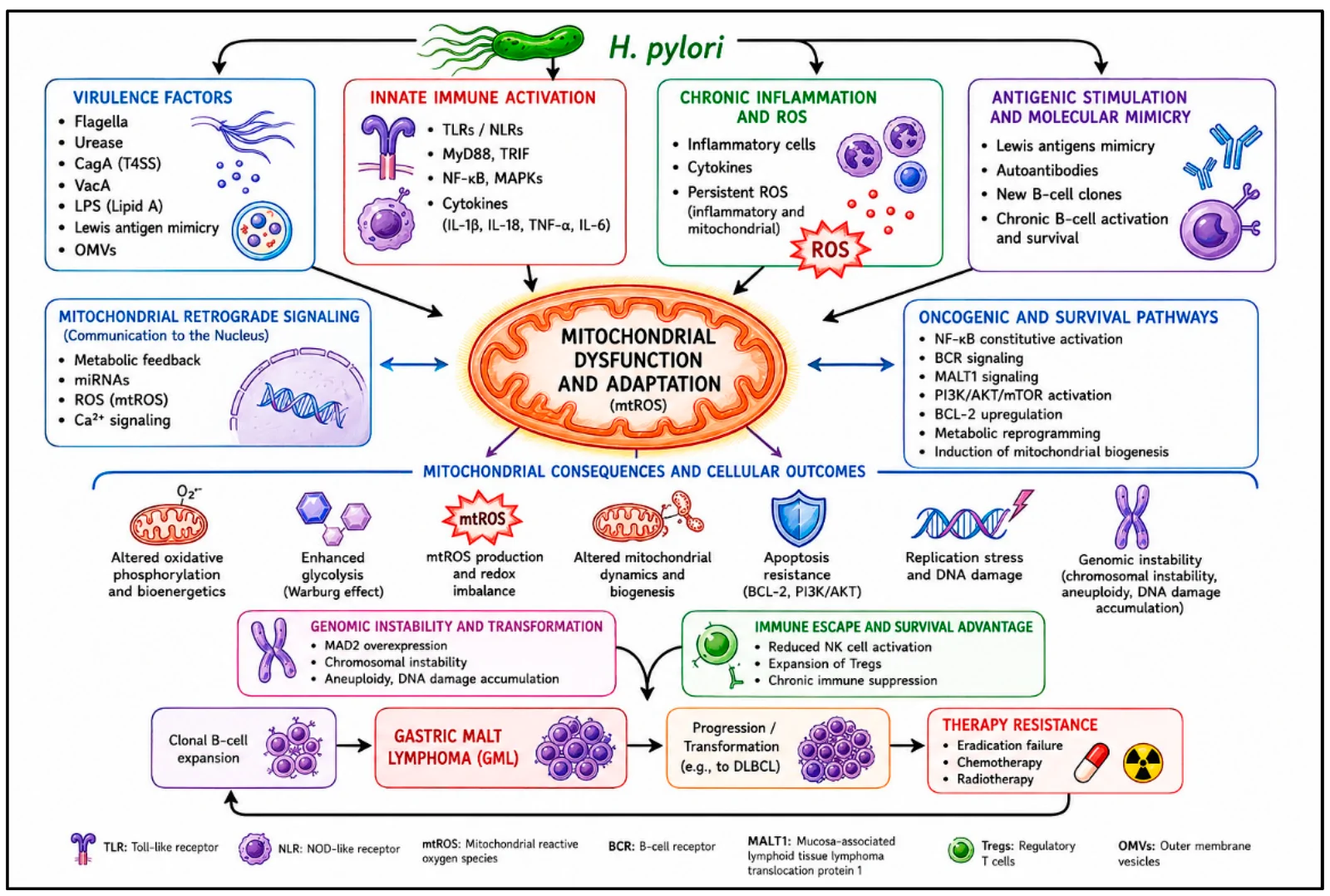
Pathogenesis of *H. Pylori*-Mediated Gastric Infection The journey from initial bacterial colonization to oncogenesis. (Left to Right): *H. pylori* adhere to the gastric epithelium and releases virulence factors (CagA, VacA, Urease) that activate host TLRs/NLRs. This triggers downstream NF-κB activation and cytokine release, driving chronic inflammation. Persistent inflammatory stress and intracellular ROS lead to mitochondrial dysfunction and metabolic reprogramming. Over time, the loss of mitochondrial-mediated apoptosis, coupled with genetic mutations and chronic antigen stimulation, drives the monoclonal expansion of B-cells, culminating in gastric MALT lymphoma (Gargiulo-Isacco Ciro).

Intracellular detection of these virulence factors and peptidoglycan fragments triggers cytosolic NLRs, promoting inflammasome assembly, caspase activation, and the secretion of pro-inflammatory cytokines such as IL-1β and IL-18 [57]. Concurrently, signaling through TLRs recruits adaptor molecules MyD88 and TRIF, activating NF-κB, mitogen-activated protein kinases (MAPKs), and interferon regulatory factors (IRFs). This persistent signaling recruits neutrophils and monocytes to the gastric mucosa, driving chronic, low-grade mucosal inflammation [58]. Reflecting a long co-evolutionary adaptation, these combined mechanisms of immune evasion and continuous antigenic stimulation prevent bacterial clearance, leading to chronic gastric inflammation. Over time, this persistent autoimmune and bacterial drive triggers the emergence and monoclonal expansion of autoreactive B-cell clones, a critical early event in the pathogenesis of GML-lymphomagenesis [59,60]. The principal mechanisms involved in *H. pylori*-mediated gastric infection are summarized in Figure 1.

## 4. Mitochondria-Centered Immunometabolic Reprogramming Induced by *H. pylori*

Although *H. pylori* employ several virulence mechanisms to establish persistent colonization of the gastric mucosa, most ultimately converge on an integrated mitochondria-centered regulatory network involving HIF-1α, STAT1, NRF2, BCL-2, and MYC [60]. Rather than acting independently, these signaling pathways are functionally interconnected through mitochondrial retrograde signaling and collectively orchestrate metabolic adaptation, redox homeostasis, immune evasion, apoptosis resistance, and cellular reprogramming. This integrated network establishes either a permissive microenvironment that supports chronic bacterial persistence, or progressively promotes lymphomagenesis and malignant transformation [60].

### 4.1. Virulence Factors and Mitochondrial Oncogenic Signaling

#### 4.1.1. CagA as the Master Regulator of Host Cell Reprogramming

Following translocation into host cells, CagA undergoes phosphorylation by Src- and Abl-family kinases, activating downstream SHP2, AKT, and ERK signaling cascades [61,62,63,64]. Rather than operating solely through classical soluble kinase pathways, intracellular CagA directly engages mitochondrial machinery, cooperating with NF-κB and ROS-mediated pathways to promote cellular survival, metabolic adaptation, and chronic inflammatory signaling [61,62,63,64].

#### 4.1.2. NF-κB Activation Is Necessary but Not Sufficient for Lymphomagenesis

However, only approximately 50% of GML cases exhibit cytoplasmic rather than nuclear localization of BCL10 and the p65 subunit of NF-κB, generally considered critical factors that lead to gastric inflammation, as no significant association has been demonstrated between the co-expression of these markers and GML development [65]. Outcomes that suggest how canonical NF-κB activation alone is inadequate to explain lymphomagenesis. Instead, a partially activated or tightly regulated NF-κB signaling state likely cooperates with mitochondrial dysfunction, metabolic remodeling, and microenvironmental alterations to sustain malignant transformation [66].

### 4.2. Innate Immune Reprogramming and Mitochondrial Stress Responses

#### 4.2.1. Suppression of Cytosolic Innate Immune Sensing

The suppression of cytosolic innate immune responses is core part of the whole infectious process toward the GML insurgence. The Retinoic acid-inducible gene I (RIG-I)—the cyclic guanosine monophosphate-adenosine monophosphate synthase (cGAS)–stimulator of interferon genes (STING) signaling pathway key parts of the body’s early defense system that find foreign genetic material from viruses and bacteria [67]. By attenuating early cytosolic pattern-recognition pathways, *H. pylori* delays effective bacterial clearance while promoting persistent colonization and establishing a state of chronic, low-grade mucosal inflammation [67,68].

As infection persists, however, progressive mitochondrial dysfunction and oxidative stress lead to the cytosolic accumulation of mtDNA, which can activate the cGAS-STING pathway independently of direct bacterial sensing. While *H. pylori* initially suppress innate immune surveillance to facilitate immune evasion, chronic mitochondrial damage progressively shifts the gastric microenvironment toward sustained sterile inflammatory signaling [67,68]. Persistent activation of the cGAS–STING pathway promotes sustained production of type I interferons and pro-inflammatory cytokines while exacerbating oxidative stress and DNA damage. This chronic inflammatory state can impair genomic integrity and promote genomic instability, creating a cellular environment conducive to lymphomagenesis [68].

During the progression to gastric mucosa-associated GML, malignant B cells frequently acquire mechanisms that attenuate or reprogram STING signaling. This adaptive rewiring allows lymphoma cells to retain the tumor-promoting benefits of chronic inflammation while minimizing the antiproliferative, immunostimulatory, and pro-apoptotic consequences of sustained innate immune activation, thereby supporting immune escape and long-term malignant cell survival [68,69].

#### 4.2.2. Mitophagy, Inflammasome Regulation, and Mitochondrial Quality Control

Host cells initially respond to mitochondrial damage by activating mitophagy, the selective removal of dysfunctional mitochondria [70,71]. However, *H. pylori* dynamically manipulate this quality-control pathway in a cell-type-dependent manner. In myeloid immune cells, early mitophagy limits mtROS-dependent inflammasome activation, easing bacterial immune evasion [72]. Conversely, chronic infection blocks autophagic flux in gastric epithelial cells and premalignant B lymphocytes, leading to the accumulation of dysfunctional mitochondria, enhanced oxidative stress, metabolic reprogramming, and genomic instability that favor survival of transformed cells [73].

The relationship between mitophagy and inflammasome activation is central to *H. pylori*-induced gastric immunometabolism [72,73,74]. While virulence factors such as CagA trigger NLRP3 inflammasome activation, efficient mitophagy normally restrains excessive inflammasome signaling by clearing damaged mitochondria and limiting mtROS accumulation [72,73,74]. Following *H. pylori* sensing (e.g., via TLR2), NLRP3 activation drives IL-1-β production, sustaining chronic mucosal inflammation [72,73,74]. However, when mitophagy becomes impaired, persistent mtROS accumulation and the release of mitochondrial danger-associated molecular patterns (mtDAMPs) continuously drive inflammasome activity. In the setting of chronic infection, this sustained inflammatory tone, cooperating with immunosuppressive Treg polarization, creates a permissive microenvironment that favors bacterial persistence, immunometabolic reprogramming, and gastric lymphomagenesis [72,73,74].

## 5. Mechanisms of *H. pylori*-Induced Gastric MALT Lymphomagenesis: The Central Role of Mitochondria

### 5.1. Mitochondria as the Integrative Hub of Chronic H. pylori Infection

Increasing evidence indicates that mitochondria constitute a central regulatory hub in the pathogenesis of GML. Beyond their well-established role in energy production, mitochondria organize cellular metabolism, oxidative stress, innate immune signaling, apoptosis, and inflammatory responses [75,76]. Many of the biological alterations induced by persistent *H. pylori* infection, including chronic inflammation, ROS production, metabolic reprogramming, defective apoptosis, and dysregulated immune signaling, also converge on mitochondrial function [75,76].

During the prolonged course of infection, which may persist for decades, *H. pylori* progressively remodel host cellular networks to establish a microenvironment that favors bacterial persistence while scattering a second host response for the survival of genetically altered cell populations [77,78]. This adaptive process involves profound mitochondrial reprogramming, enabling infected and surrounding cells to maintain bioenergetic flexibility despite sustained oxidative and metabolic stress [78].

Alterations in mitochondrial metabolism influence both epithelial and immune cell behavior, promoting metabolic plasticity, immune evasion, resistance to apoptosis, and progressive clonal evolution [76]. The following sections explore how these mitochondrial adaptations contribute to the initiation, maintenance, and eventual progression of GML.

### 5.2. Hypoxia, Oxidative Stress, and Metabolic Reprogramming

Although only a small proportion of *H. pylori* infections progress to GML, chronic infection alters the tissue microenvironment through relative hypoxia, oxidative stress, extracellular acidification, and metabolic instability [79,80]. This severe microenvironment creates a selective pressure that favors metabolically flexible cellular clones capable of adapting to energetic stress while resisting apoptosis. Supporting this adaptive model, the predominantly cytoplasmic localization of BCL10 and NF-κB p65 in a subset of GML suggests a distinct signaling mode driven by persistent mitochondrial stress and inflammation rather than simple constitutive nuclear NF-κB activation [81].

At this stage, mitochondrial dysfunction functions as an active driver of malignant progression rather than a passive byproduct of inflammation [82,83]. *H. pylori* virulence factors (particularly CagA) and chronic mtROS generation stabilize HIF-1α, establishing a state of pseudohypoxia under normoxic conditions while simultaneously suppressing p53 and activating JAK/STAT signaling [84,85]. These convergent signals rewire cellular bioenergetics toward glycolytic reliance, extracellular acidification, and angiogenesis [86].

Crucially, while *H. pylori* initiates this metabolic reprogramming, long-term maintenance of the transformed phenotype shifts to somatic mtDNA mutations. Lacking protective histones, located near the electron transport chain (ETC), and possessing limited repair mechanisms, mtDNA is particularly vulnerable to chronic, inflammation-derived oxidative damage during antigen-driven B-cell expansion [87,88,89]. In turn, these somatic mtDNA mutations alter electron transport efficiency, perpetuate ROS generation, and inhibit prolyl hydroxylases (PHDs), thus locking in HIF-1α and STAT-3 stabilization and maintaining pseudohypoxia independently of bacterial presence [90,91,92].

This self-sustaining adaptive state operates via a metabolic “Goldilocks effect”, in which ROS levels remain sufficiently elevated to drive pro-survival, pro-proliferative, and inflammatory signaling, yet low enough to avoid triggering irreversible bioenergetic collapse or apoptosis [93,94,95]. Clonal selection favors mild heteroplasmic mtDNA mutations (particularly in complexes I and III) that strike this precise balance, preserving sufficient ATP synthesis for proliferation while eliminating severe mutations through negative selection. Ultimately, this progressive transition from bacterial trigger to mitochondrial genomic instability explains how GML evolves from an antigen-dependent tumor into an autonomous, antigen-independent malignancy capable of persisting post-*H. pylori* eradication [96,97].

### 5.3. Hypoxia-Responsive and Epigenetic microRNAs Reinforce Mitochondrial Adaptation and Cell Fate

The establishment of pseudohypoxia and cellular plasticity during chronic *H. pylori* infection is heavily reinforced by epigenetic mechanisms involving microRNAs (miRNAs) [98]. Beyond immediate host response, *H. pylori* infection drives the upregulation of specific microRNAs, notably miR-210, miR-584, and miR-1290, which are closely linked to HIF-1α and STAT-3 signaling pathways, mitochondrial adaptation, and tumor progression [99]. These miRNAs participate in integrated transcriptional networks that coordinate cellular responses to chronic hypoxia, oxidative stress, and metabolic remodeling [98,100,101,102].

In parallel with hypoxia-driven signaling, epigenetic control extends to lineage plasticity and differentiation programs [33]. Increased expression of miR-200 and miR-210 exerts a protective, regulatory effect by directly targeting and inhibiting the zinc-finger transcription factor ZEB1 [33]. Operating alongside elevated BCL-6 expression, this miR-200/210–ZEB1 axis functions to constrain aberrant epithelial-to-mesenchymal transition (EMT) and preserve cell identity during persistent inflammation [33].

However, under sustained stress, downstream suppressive mechanisms predominate. One critical consequence is the suppression of the tumor suppressor transcription factor FOXD3, whose expression is frequently reduced in *H. pylori*-associated gastric lesions through both miRNA-mediated silencing and promoter hypermethylation [103]. Loss of FOXD3 disrupts normal differentiation programs while favoring cellular plasticity and survival under persistent inflammatory stress, facilitating early neoplastic transformation [103].

Beyond their canonical cytoplasmic functions, these hypoxia-responsive miRNAs have also been detected within mitochondria, where they function as mitomiRs capable of directly regulating mitochondrial gene expression [104]. By modulating OXPHOS, ROS homeostasis, mitochondrial biogenesis, and metabolic flexibility, mitomiRs provide an additional regulatory layer that stabilizes the metabolically reprogrammed phenotype, earlier induced by chronic *H. pylori* infection [104,105,106].

Collectively, these epigenetic mechanisms complement mitochondrial genomic evolution by reinforcing oxidative stress tolerance, maintaining pseudohypoxic signaling, and preserving the metabolic plasticity required for continued clonal expansion. Therefore, rather than initiating malignant transformation independently, hypoxia-responsive and lineage-regulating miRNAs appear to consolidate the adaptive mitochondrial phenotype established during chronic infection, facilitating progression toward lymphomagenesis [104].

### 5.4. Proposed Mitochondrial Transfer as a Mechanism of Metabolic Rescue

Beyond the established paracrine effects of cytokines and growth factors, intercellular mitochondrial transfer may represent an additional mechanism of metabolic communication within the gastric lymphoma microenvironment. We proposed that mitochondrial transfer from M2-polarized macrophages and stromal cells, including cancer-associated fibroblasts (CAFs), to chronically stimulated B lymphocytes could provide an early adaptive mechanism supporting clonal survival during lymphomagenesis [107].

Although this phenomenon has not been directly demonstrated in GML, evidence from solid tumors and hematological malignancies indicates that stromal cells can transfer functional mitochondria to metabolically stressed neighboring cells [108]. Such exchange has been associated with restoration of OXPHOS, increased ATP production, improved bioenergetic capacity, and reduced susceptibility to apoptosis [108,109].

This mechanism could be particularly relevant within the chronically inflamed gastric mucosa, where persistent *H. pylori* stimulation, oxidative stress, and fluctuating oxygen availability impose substantial metabolic demands on antigen-stimulated B cells [110,111,112]. If mitochondrial transfer occurs in this setting, the acquisition of functional mitochondria could transiently restore respiratory capacity, improve redox homeostasis, and maintain cellular viability during repeated cycles of antigenic stimulation.

Intercellular mitochondrial exchange can also occur through extracellular vesicles, mitochondria-containing vesicles, and tunneling nanotubes (TNTs), dynamic actin-based structures capable of transferring intact organelles between neighboring cells [113,114,115]. Mitochondrial trafficking through TNT-like structures has also been described in malignant B-cell models, providing biological plausibility for a similar mechanism in GML, although direct evidence in this disease remains lacking [113,114,115].

We therefore suggested that M2 macrophages and CAFs may function as potential metabolic donors within the chronically inflamed gastric microenvironment, providing mitochondria to metabolically stressed B-cell clones [116,117]. Such transfer could transiently restore OXPHOS, limit excessive ROS accumulation, preserve ATP production, and reduce mitochondrial apoptotic susceptibility. Rather than acting as a primary driver, stromal mitochondrial transfer may provide a crucial survival bridge. By transiently preserving the viability of metabolically strained, genetically unstable B-cell clones, this microenvironmental support could extend their lifespan, allowing sufficient time for secondary genetic and epigenetic alterations to accumulate, ultimately driving progression toward autonomous lymphoma [116,117].

### 5.5. Mitochondrial Dynamics and Clonal Progression Toward Autonomous GML

As GML progresses from an antigen-dependent lesion to an autonomous neoplasm, mitochondrial remodeling extends beyond metabolic reprogramming to include profound alterations in organelle dynamics [118,119]. Mitochondria continuously undergo fusion and fission, two highly coordinated processes that regulate mitochondrial quality control, bioenergetic efficiency, calcium homeostasis, and cellular adaptation to stress. In malignant cells, however, this balance progressively shifts toward excessive mitochondrial fragmentation, reflecting an adaptive response to the increasing metabolic and proliferative demands of the evolving lymphoma clone [119].

A hallmark of this steady adaptation pathway is the increased activity of the mitochondrial fission machinery, particularly dynamin-related protein 1 (DRP1) and mitochondrial fission protein 1 (FIS1) [120]. Enhanced DRP1-mediated fission generates smaller, highly mobile mitochondrial fragments that can be rapidly redistributed throughout the cytoplasm according to localized energetic requirements [119,120]. Beyond regulating mitochondrial morphology, this process optimizes ATP delivery and calcium buffering at sites of increased metabolic demand, favoring cytoskeletal remodeling, cell migration, and adaptation to a chronically inflamed microenvironment [119,120].

As additional genetic and epigenetic alterations accumulate, these metabolically adapted B cell clones progressively lose their dependence on chronic *H. pylori*-driven antigenic stimulation and acquire increasingly autonomous growth characteristics [89]. Consequently, mitochondrial dynamics should be regarded not merely as structural changes accompanying malignant transformation but as active determinants of disease progression [121,122]. By integrating bioenergetic adaptation with enhanced cellular survival and proliferative capacity, mitochondrial fission represents the final step in a continuum that begins with *H. pylori*-induced mitochondrial dysfunction and culminates in the emergence of autonomous lymphoma [121,122]. This progressive remodeling establishes the biological foundation for the more advanced mitochondrial adaptive programs, including antioxidant reprogramming, mitochondrial retrograde signaling, and therapeutic vulnerabilities, that are discussed in the following chapter.

## 6. Mitochondria at the Crossroads of *H. pylori* Persistence, Immunometabolic Reprogramming, in GML-Genesis

From a broader and clinically relevant perspective, *H. pylori* may be more accurately viewed not as a direct mutagenic carcinogen, but as a chronic ecological stressor that continuously induce remodeling the gastric microenvironment. Over decades of chronic inflammation, these mitochondrial adaptive mechanisms that ultimately drive to malignant transformation might be seen as unique defensive response [122].

Interestingly, once malignant transformation is established, *H. pylori* often decline or disappears from the gastric mucosa. This phenomenon, known as a “hostile takeover,” reflects the profound microenvironmental remodeling initially triggered by chronic infection but subsequently sustained by tumor progression [123,124]. Chronic gastritis promotes glandular atrophy, hypochlorhydria, and architectural distortion, creating a gastric niche that paradoxically no longer supports persistent *H. pylori* colonization, despite the bacterium having initiated the oncogenic process [123,124].

### 6.1. The MYC, NRF2, and BCL-2 Survival Axis

Fundamentally, this metabolic remodeling extends well beyond the classical Warburg paradigm of aerobic glycolysis, representing an integrated redox–biosynthetic adaptation orchestrated by a cooperative NRF2 and MYC network [125]. Under persistent, sublethal mtROS exposure, NRF2 coordinates antioxidant responses while MYC drives mitochondrial biogenesis to support rapid proliferation [37,126]. To tolerate this hyperactive state, these pathways sustain BCL-2 expression at the outer mitochondrial membrane [126,127]. By preserving membrane integrity and preventing mitochondrial outer membrane permeabilization (MOMP) and cytochrome *c* release, BCL-2 raises the apoptotic threshold [37,127,128]. Together, MYC-driven metabolic reprogramming and BCL-2-mediated survival signaling establish the permissive environment required for malignant transformation, clonal expansion, and disease progression [38,39].

Because BCL-2 does not protect against iron-dependent lipid peroxidation, NRF2 simultaneously induces a potent anti-ferroptotic defense program by upregulating GPX4 and glutathione (GSH) synthesis enzymes [129]. This anti-ferroptotic machinery is tightly coupled to mitochondrial metabolism, as regenerating reduced GSH requires a continuous supply of NADPH/NADH maintained via nicotinamide nucleotide transhydrogenase (NNT) and tricarboxylic acid (TCA) cycle activity [130].

By preserving residual respiratory chain function, the malignant clone sustains the reducing power required to suppress ferroptosis while simultaneously channeling TCA cycle intermediates into nucleotide biosynthesis. Importantly, intact mitochondrial electron transport chain (ETC) activity remains indispensable not primarily for ATP generation, but for maintaining the oxidation of NADH to NAD+ [129,130]. This NAD+ pool maintains uninhibited TCA cycle flux and oxaloacetate production. Oxaloacetate is subsequently converted into aspartate, a rate-limiting precursor for de novo pyrimidine/purine synthesis, RNA transcription, and protein synthesis. In chronic *H. pylori*-driven B-cell expansion, this mitochondrial node provides the essential metabolic foundation to support the heavy biosynthetic demands imposed by MYC [129,130].

### 6.2. The Evolutionary Imperative of Mitochondria and the Transition to Antigen Independence of Fully Functionally Cancer Stem Cells

GML reflects a progressive metabolic and molecular evolution [131]. In its early phases, continuous *H. pylori*-dependent antigenic stimulation drives the expansion and selection of reactive B-cell populations [131]. During later stages, GML acquires sequential genetic and epigenetic alterations, including BIRC3–MALT1 translocations, retrotransposon activation, and BCL10 mutations, that drive constitutive NF-κB activation and transition the tumor toward antigen independence [132]. This evolution raises a fundamental question: why do mitochondria emerge as central orchestrators during this late transformation phase? The answer lies in their evolutionary origin as ancestral α-proteobacterial endosymbionts [133].

Mitochondria retain their own genome and an extensive retrograde signaling capacity that enables continuous communication with the nucleus [134]. During chronic *H. pylori* infection, rather than suffering bioenergetic failure, mitochondria adaptively remodel their function under persistent microenvironmental stress—analogous to bacterial metabolic adaptations under antibiotic or nutritional pressure [134]. Stressed mitochondria emit a continuous stream of retrograde signals, including mtROS, TCA cycle intermediates, calcium fluxes, and mitochondrial danger-associated molecular patterns (mtDAMPs), that fundamentally reshape nuclear transcription [135].

Through this persistent retrograde signaling (including LINE1 retrotransposon activation), mitochondria transition from passive targets of infection to active manipulators of cellular adaptation [136,137]. Pathways involving MYC, STAT3, and HIF-1α become sustained, locking B-cell clones into a self-sustaining phenotype capable of surviving independently of the initiating bacterial stimulus [136,138]. While MYC overexpression sustains the hypermetabolic state required for rapid expansion, it also increases apoptotic priming [138,139]. To maintain viability, the transformed B-cell clone relies on mitochondrial anti-apoptotic mechanisms, specifically BCL-2-mediated neutralization of BH3-only proteins and suppression of MOMP-induced cytosolic release of SMAC/DIABLO [136,139].

Crucially, altered mitochondrial TCA cycle flux generates metabolites (such as α-ketoglutarate, succinate, and fumarate) capable of regulating chromatin-modifying enzymes, thereby reshaping nuclear epigenetic landscapes [140]. This retrograde chromatin remodeling fosters the aberrant reactivation of developmental and pluripotency-associated networks, including NANOG, SOX2, and OCT4 [140]. Once established, this epigenetic reprogramming drives a process of dedifferentiation toward a multipotent, stem-like state, conferring self-renewal capacity, phenotypic plasticity, and resistance to therapeutic stress [141]. At this critical juncture, *H. pylori* has driven the system across a threshold into a new homeostatic equilibrium: the transformed B-cell clones are no longer reliant on microbial stimulation, but have acquired an autonomous multipotent stem-like state capable of sustaining their malignant phenotype [142]. 

### 6.3. A Targeted Therapeutic Vulnerability

**Figure 2:**
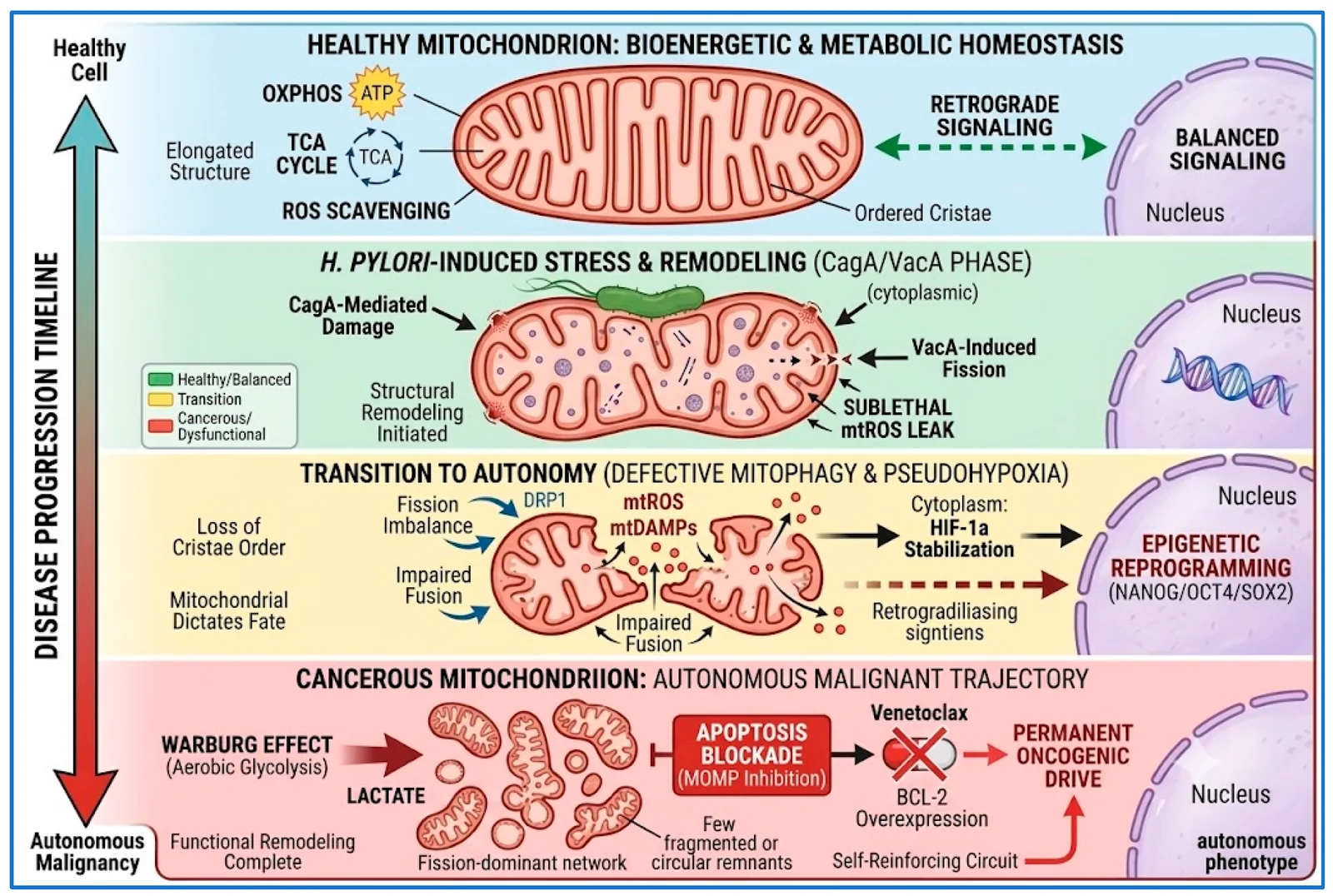
Mitochondrial Remodeling Timeline in *H. pylori* Oncogenesis. Healthy State: Mitochondria show a normal, elongated ultrastructure with tightly packed, parallel cristae, maintaining physiological OXPHOS, TCA cycle activity, and homeostatic retrograde signaling with the host cell nucleus. Stress and Remodeling Phase: Delivery of bacterial oncoproteins (CagA/VacA) induces initial mitochondrial swelling, cristae disorganization, and a sublethal leak of mitochondrial ROS (mtROS). Transition to Autonomy: An altered fission/fusion balance (DRP1-driven) fragments the network. Defective mitophagy prevents the clearance of these damaged organelles, leading to chronic mtROS/mtDAMP leakage that stabilizes HIF-1α and induces nuclear epigenetic reprogramming (NANOG, SOX2, OCT4). Malignant Autonomy: Transformed mitochondria become highly fragmented, swollen spheres with concentric or circular cristae remnants. Cells adapt via the Warburg effect and upregulate BCL-2 to block apoptosis, locking in an antigen-independent, autonomous malignant phenotype susceptible to BH3 mimetics (Venetoclax) (Gargiulo-Isacco Ciro).

The very adaptations that confer stress tolerance may also generate a therapeutic dependency on the mechanisms that maintain mitochondrial integrity and suppress apoptosis [143,144]. The use of BH3 mimetics, particularly the selective BCL-2 inhibitor venetoclax, therefore represents a rational mitochondria-targeted strategy to disrupt this protective equilibrium. By releasing pro-apoptotic effectors from BCL-2 and favoring MOMP, venetoclax can overcome the apoptotic threshold of cells that have become highly adapted to persistent metabolic and oxidative stress. This vulnerability may be particularly relevant during the transition from *H. pylori*-driven cellular stress to increasingly autonomous lymphoma growth, when mitochondrial adaptation becomes a critical determinant of malignant cell survival [145] (Figure 2).

Thus, the same mitochondrial plasticity that enables B-cell clones to withstand chronic microbial pressure may ultimately become their Achilles’ heel. The progressive remodeling of mitochondrial metabolism, redox control, and apoptotic signaling creates a state of acquired fitness that is accompanied by a corresponding therapeutic liability [146]. Targeting this liability may therefore provide an opportunity to intervene not simply against the lymphoma cell, but against the mitochondrial adaptations that allowed it to survive, evolve, and ultimately escape the constraints of its original microbial environment [147].

## 7. Conclusions

Collectively, these findings suggest that mitochondrial dysfunction is not merely a consequence of chronic infection, but rather an integrative combination linking microbial persistence, inflammatory signaling, metabolic adaptation, and neoplastic evolution. Through bidirectional communication between mitochondria and the nucleus, alterations in OXPHOS, glycolysis, ROS homeostasis, and mitochondrial architecture drive genomic instability, immune evasion, a multipotent stem-like phenotype, and disease progression. Thus, alongside established genetic and inflammatory pathways, metabolic and microenvironmental selective pressures play a fundamental role in the pathogenesis and evolution of GML. From an evolutionary perspective, mitochondria emerge as key determinants of cellular adaptation and clonal survival during chronic *H. pylori*-driven inflammation. By continuously adapting cellular bioenergetics and suppressing stress-induced apoptosis, mitochondria drive the creation of dedifferentiated multipotent cancer stem cells generating strong resilient clonal populations. Over time, these adaptations contribute to the emergence of lymphoma cells endowed with exceptional survival capacity, enabling them to evade immune clearance, tolerate metabolic stress, and develop resistance to standard chemo- and radiotherapy.

## Figures and Tables

**Table 1:** Antigenic components of *H. pylori*.

Antigenic Component of *H. pylori*	Structural/Molecular Characteristics	Biological and Immunological Effects
*H. pylori* Lipid A (tetra-acyl Lipid A)	Characterized by long acyl chains containing 16–18 carbon atoms, making the overall LPS less toxic compared with Lipid A from other Gram-negative bacteria.	Reduced endotoxic activity; contributes to immune modulation and persistence of chronic infection.
*H. pylori* Lipid A Immunomodulation	Weak activator of innate immune signaling pathways.	Weakly activates natural killer (NK) cells and suppresses T regulatory (Treg) cells, altering host immune responses.
O-polysaccharide Chain of LPS	Highly fucosylated structure that mimics human Lewis antigens and H-1 antigen.	Molecular mimicry promotes autoantibody production and emergence of novel B-cell clones, contributing to immune dysregulation and chronic inflammation.
CagA (Cytotoxin-associated gene A)	Expressed in approximately 70% of *H. pylori* strains; translocated into host cells through the Type IV Secretion System (T4SS).	Promotes chronic gastric infection, epithelial signaling dysregulation, inflammation, and tumorigenesis.
VacA (Vacuolating cytotoxin A)	Pore-forming toxin capable of receptor binding on host cells.	Induces cellular injury, immune modulation, chronic gastric infection, and contributes to tumorigenesis.
Outer Membrane Vesicles (OMVs)	Membrane-derived vesicles containing bacterial toxins, enzymes, and signaling molecules.	Facilitate immune evasion, sustain chronic gastric inflammation, and promote oncogenic processes.

**Table 2:** Pathogenesis of *H. pylori* mediated gastric infection and lymphoma.

Phase	Key Drivers and Virulence Factors	Molecular and Metabolic Mechanisms	Pathological Outcome (GML Progression)
1. Colonization and Innate Evasion	Urease Flagella Tetra-acylated Lipid A Lewis antigen mimicry	Buffers stomach acid with ammonia. Weakly activates TLRs to evade immune detection. Provokes autoantibodies via mimicry, triggering early B-cell recruitment.	Establishment of persistent bacterial colonization within the gastric mucus layer.
2. Chronic Inflammation and Stress	CagA and VacA oncoproteins Th17-to-Treg cell shift IL-8, IL-17, and IL-10	CagA activates proliferative pathways (Src, ERK, AKT) and suppresses p53. VacA impairs mitophagy, leading to the accumulation of damaged mitochondria. Shift to Treg cells suppresses host antitumor immunity.	Chronic inflammatory microenvironment with progressive host cellular injury.
3. Mitochondrial Reprogramming	Mitochondrial ROS (mtROS) HIF-1α stabilization	High mtROS induces somatic mutations in mitochondrial DNA. Stabilization of HIF-1α initiates a “pseudohypoxic” metabolic shift toward aerobic glycolysis.	Cellular adaptation to oxidative stress and initiation of metabolic rewiring.
4. Clonal Selection and Autonomy *(The NRF2–MYC Axis)*	NRF2 activation MYC oncogene expression M2 Macrophages (BAFF/APRIL) BCL-2 upregulation SMAC/DIABLO suppression	NRF2 drives the antioxidant response, neutralizing lethal ROS and protecting mutated clones. NRF2 and MYC cooperate to reprogram metabolism (pentose phosphate pathway) for rapid cell growth. MYC overexpression lowers the threshold for mitochondrial apoptosis while increasing metabolic stress. Upregulation of BCL-2 prevents outer mitochondrial membrane permeabilization, blocking apoptosis.	Monoclonal Gastric MALT Lymphoma (GML/MALToma): Highly resilient, chemotherapy-resistant, and antigen-independent B-cell malignancy.

## Data Availability

Data can be asked to the authors if needed.
